# A cylindrical assembly model and dynamics of the Ebola virus VP40 structural matrix

**DOI:** 10.1038/s41598-018-28077-7

**Published:** 2018-06-27

**Authors:** Elumalai Pavadai, Bernard S. Gerstman, Prem P. Chapagain

**Affiliations:** 1 Department of Physics, Florida International University, Miami, Florida 33199 USA; 2 Biomolecular Sciences Institute, Florida International University, Miami, Florida 33199 USA

## Abstract

The Ebola filovirus causes severe hemorrhagic fever with a high fatality rate in humans. The primary structural matrix protein VP40 displays transformer-protein characteristics and exists in different conformational and oligomeric states. VP40 plays crucial roles in viral assembly and budding at the plasma membrane of the infected cells and is capable of forming virus-like particles without the need for other Ebola proteins. However, no experimental three-dimensional structure for any filovirus VP40 cylindrical assembly matrix is currently available. Here, we use a protein-protein docking approach to develop cylindrical assembly models for an Ebola virion and also for a smaller structural matrix that does not contain genetic material. These models match well with the 2D averages of cryo-electron tomograms of the authentic virion. We also used all-atom molecular dynamics simulations to investigate the stability and dynamics of the cylindrical models and the interactions between the side-by-side hexamers to determine the amino acid residues that are especially important for stabilizing the hexamers in the cylindrical ring configuration matrix assembly. Our models provide helpful information to better understand the assembly processes of filoviruses and such structural studies may also lead to the design and development of antiviral drugs.

## Introduction

The Ebola virus disease causes severe hemorrhagic fever with a high fatality rate in humans and is especially dangerous because of a lack of effective vaccines^1,2^. This disease, transmitted by human-to-human contact, is caused by four different Ebola viruses that were originally transmitted to humans from wild animals. Of the four Ebola viruses, the Ebola virus and Sudan virus are responsible for most of the Ebola clinical cases^3,4^. A better understanding of the molecular mechanisms responsible for Ebola virus replication is an essential step in the development of antiviral drugs.

**Figure 1:**
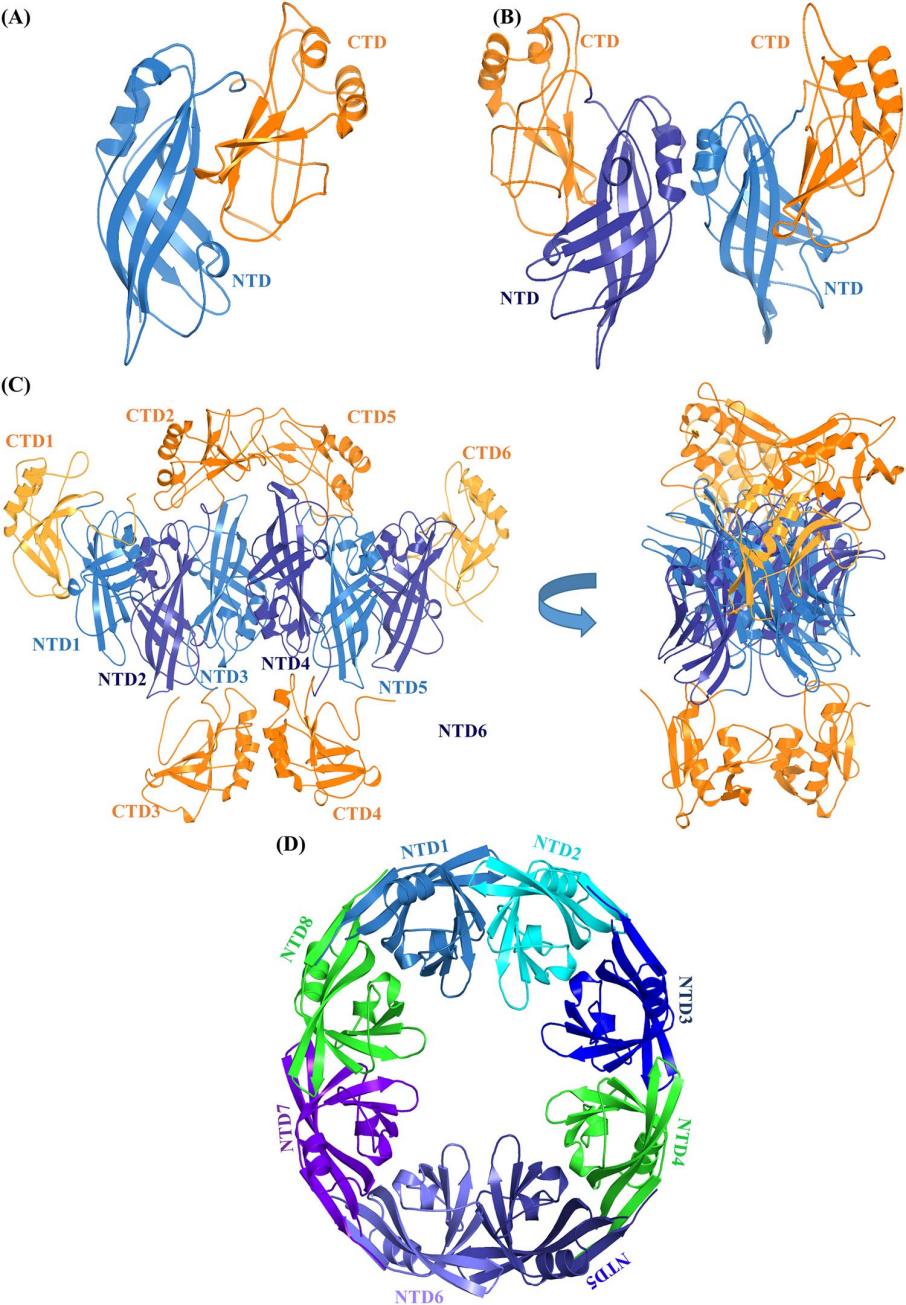
Different conformational states of VP40. (**A**) Monomeric^10^, (**B**) dimeric^14^, (**C**) hexameric^14^ and (**D**) octameric structures of VP40^14^. In (**C**), the four CTDs (2, 3, 4 and 5) missing in the crystal structure that were added using Modeller software are shown in orange color, and the hexamer is rotated 90° along the vertical-axis to provide a view that clarifies the position of the missing CTDs. “Sprung” CTD2 and CTD5 could form the outer viral matrix while sprung CTD3 and CTD4 could form the inner matrix^14^.

A single-stranded, negative-sense RNA genome in the Ebola virus encodes a relatively small number of proteins, some of which perform multiple functions^2^. The 40 kDa, 326 amino acid protein VP40 is the primary structural matrix protein that creates the scaffolding for the budding of the host membrane that produces infectious virus particles^5–9^. VP40 has a distinctive N-terminal domain (NTD) and C-terminal domain (CTD) (Fig. 1A). The NTD and CTD have a limited number of inter-domain contacts, which provides the flexibility for rearrangement of the NTD and CTD relative to each other to form different conformational states as shown in Fig. 1^10–12^. This conformational flexibility allows VP40 to display transformer-protein characteristics^13^ by arranging into different configurations to perform different functions in the virus life cycle^6,14^: a butterfly-shaped dimer structure (Fig. 1B) that is essential for membrane trafficking^15^, a hexameric structure (Fig. 1C) that acts as a structural building block of the cylindrical viral matrix filament^14^, and an RNA-binding octameric ring structure (Fig. 1D) that controls viral transcription^11,14^. Figure 1C,D show that upon oligomerization, some CTDs can dissociate or become ‘sprung’ from their NTDs. However, the molecular mechanisms responsible for these structural transformations are not yet clear.

VP40 expression in eukaryotic cells shows that VP40 can independently assemble into and release filamentous virus-like particles (VLPs)^16–18^. In addition, the co-expression of glycoprotein (GP) and/or nucleoprotein (NP) along with VP40 significantly enhances VLP release^17^. These observations demonstrate that VP40 is essential for the formation of the viral matrix. In addition, X-ray crystallography^14^, cryo-electron microscopy (cryo-EM) and electron 2D-tomography (cryo-TM) studies^19,20^ have demonstrated that the VP40 matrix consists of long, linear arrangements of hexamers arranged into filaments in the cylindrical lattice^14^. However, how the VP40 filaments assemble into the cylindrical lattice has remained elusive.

To better understand the cylindrical assembly of the VP40 matrix protein and its structural role in the Ebola virion, we have modeled the cylindrical assembly of VP40 using computational techniques. Based upon experimental measurements of the size of Ebola virus particles, we used protein-protein docking computational techniques to create cylindrical models of 42 and 92 nm^19^ diameters with varying numbers of VP40 hexamers for each size. The 92 nm ring represents the size of an active Ebola virion, whereas the 42 nm ring represents the observed Ebola structure that does not contain genetic material. We then chose a subset of these models that we selected based on a set of criteria described below, including consistency with the electron 2D average tomograms of the Ebola virion. We analyzed the interactions and arrangements within a pair of hexamers from the energetically stable models to determine which amino acid residues are especially important for stabilizing the cylindrical matrix assembly. We also used all-atom molecular dynamics simulations to optimize the structures of the cylindrical models and investigate their short-term dynamics. Our models provide helpful information to better understand the assembly processes of filoviruses and such structural studies may also lead to the design and development of antiviral drugs.

## Results

### Construction of the cylindrical VP40 matrix

Previous studies have shown that VP40 is flexible, and that it can assemble into multi-conformational states (Fig. 1). Our specific aim is to investigate the dynamics of formation and structural stability of the Ebola VP40 cylindrical ring matrix. The VP40 monomer assembles into a dimer through NTD-NTD interactions in solution, and VP40 dimers assemble into a hexamer through multiple steps as explained in ref.^14^, and the VP40 hexamers further assemble end-to-end into linear filaments through CTD-CTD interactions. Bornholdt *et al*.^14^ showed experimentally that in the Ebola virion filament assembly, the NTDs and end CTDs (1 and 6 in Fig. 1C) of the hexamer form the filament’s main structural matrix, while the sprung up CTDs (2 and 5) and down CTDs (3 and 4) form upper (outer) and lower (inner) structural layers, respectively. The upper CTD matrix faces toward the viral lipid membrane while the lower matrix faces inward toward nucleocapsid proteins^6,14^. In addition, the 3D-reconstruction study also showed that the VP40 hexamer ring structures assembled *in vitro* in a similar manner, forming the upper and lower CTD matrices^10^.

In our study, the cylindrical models are created and ranked by SymmDock according to a geometric shape complementarity score. Top ranked docking complexes from each of the symmetric orders was visually inspected, and the orientation and interactions between hexamers in the cylindrical complexes were analyzed. For this, a set of criteria was developed based on comparison to experimentally observed hexamer and linear filament structures of VP40^14^, and cryo-EM and cryo-TM of VP40 of the Ebola virion^19–21^. Recent cryo-EM and cryo-TM studies of the Ebola virion observed that without a nucleocapsid, the VP40 VLPs have a wavy cylindrical filamentous envelope with an irregular diameter of 48–52 nm, while the authentic VLPs with a nucleocapsid have a regular filamentous cylindrical envelop with a diameter of 96–98 nm. In addition, these cryo-EM and cryo-TM studies have indicated that the VP40 matrix arranges in a cylindrical lattice with 5 nm periodicity spacing between the hexamers around the ring in both of these VP40 cylindrical assemblies^19,20^. Importantly, a linear filament structure containing three hexamers arranged end-to-end based on the X-ray protein crystallography has been shown to align well onto the electron 2D average tomograms of VP40 of the authentic virion, signifying that the VP40 matrix consists of linear hexamers arranged end-to-end into filaments in a cylindrical lattice^14^.

Based on this information, VP40 cylindrical docking complexes that were ranked highly by SymmDock were selected for further analyses using the following criteria: (i) The cylindrical docking ring models should have outer diameters of at least 42 nm (empty VP40 cylindrical matrix without nucleocapsid) or 92 nm (VP40 cylindrical matrix with nucleocapsid). The diameter of the VP40 rings are smaller than the actual filaments because the total thickness of the viral lipid membrane combined from both sides is 6 nm (3 nm at one end and 3 nm on the other end of the diameter of the viral cylinder), and this 6 nm is subtracted from the original 48 and 98 nm diameters of the cylindrical VP40 matrix. (ii) CTDs 2 and 5 of the VP40 hexamers should face outward from the cylindrical complex towards the viral lipid membrane, and CTDs 3 and 4 should face inward toward the nucleocapsid. (iii) The VP40 filament of the cylindrical docking models should align onto the electron 2D average tomograms of VP40 of the authentic virion so that additional hexamers can be added end-to-end to form filaments.

Using these criteria, we selected specific SymmDock complexes for further investigation of symmetric order 21, 22, and 23 for the 42 nm diameter ring, and 52, 53 and 54 symmetric order for the 92 nm ring complex. SymmDock parameters for these complexes are presented in Table 1. The analysis of Table 1 indicates that, as expected, higher shape complementarity and larger interaction area between side-by-side hexamers give the best orientations. To increase the possibility of finding good cylindrical models, additional docking calculations were performed using alternative initial orientations of the CTDs that are missing from the crystal structure. From these additional docking models, those that fit the criteria given above for the 42 nm diameter rings have VP40 orientations and scores very similar to the models listed in Table 1 and thus were not treated separately. However, one of the additional docking models for the 92 nm diameter ring with 54 VP40 hexamers [entry 54(b)] displayed a reasonable orientation but with a more stable energy, and this model was added to Table 1 for further study. As shown in Table 1, all these models have negative atomic contact energy (ACE) values^22^, indicating that the VP40 hexamers assemble into a cylindrical ring matrix with substantial contribution from the hydrophobic regions of the hexamers.

**Table 1:** Selected docking complexes and their symmetry order, geometry score, interface area and atomic contact energy from SymmDock.

Symmetry order^a^	Docking score^b^	Interface area (Å^2^)^c^	Atomic contact energy (ACE) (kcal/mol)^d^
21	11554	3235.20	−484.55
22	12442	2743.50	−272.66
23	12808	2911.90	−342.09
52	13374	3441.40	−138.88
53	13568	3474.80	−147.07
54 (a)	13712	3500.40	−150.60
54 (b)^*^	11044	1951.90	−382.54

^a^Number of VP40 hexamers in the docking ring complex. ^b^Geometric shape complementarity score that SymmDock uses to rank the complexes. ^c^Approximate interface area of the complex. ^d^ACE: <0 indicates an hydrophobic environment and ACE > 0 indicates hydrophilic or solvent exposed environments^22^. ^*^Docking model using an initial hexamer structure with alternative orientations of the four CTDs missing from the X-ray structure.

**Figure 2:**
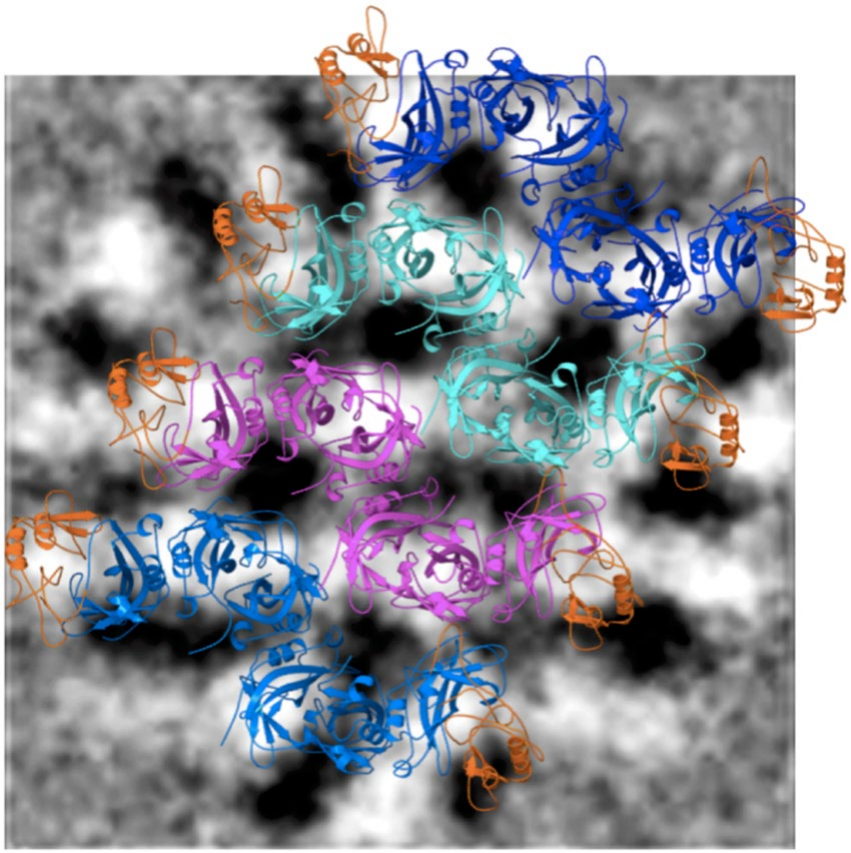
A portion of the VP40 cylindrical ring matrix of model 54(b) showing four hexamers side-by-side, superimposed onto 2D averages of virion tomograms. The tomograms are adapted from ref.^19^ and black color has been inverted to white for clarity in this image; white corresponds to VP40 density. As in ref.^14^, we overlay the side-by-side core model onto the 2D virion tomograms of VP40 for model validation. The proteins are displayed as ribbon diagrams. The two CTDs (one at each end) of each hexamer that are part of the cylindrical matrix and present in the X-ray structure are colored as orange. The six NTDs are colored differently for each of the four hexamers: light blue, magenta, cyan, or dark blue.

In order to further ensure the selection of energetically stable ring models for the 42 and 92 nm diameter cylindrical VP40 matrix, pairwise interaction energy (interaction between side-by-side hexamers) and hydrogen bond analyses averaged over all hexamer pairs were performed on the energy minimized rings. The results are shown in Table 2. Cylindrical model 21 shows the strongest average interaction energy of −303.56 kcal/mol and the most number of hydrogen-bonds of 12.57 compared to other models of the 42 nm diameter cylinder. The cylindrical model 54(b) displays the strongest average interaction energy of −301.05 kcal/mol and the largest number of hydrogen-bonds of 10 compared to other models of the 92 nm diameter cylinder. This suggests that the 21 and 54(b) models have especially favorable hexamer-hexamer interactions in the cylindrical assembly that may be due to better arrangements of hexamers within the cylinders, such as inter-hexamer distance (Table 2). As shown in Fig. 2, superimposition of the side-by-side layout of four hexamers from 54(b) onto 2D averages of virion tomograms (adapted from Beniac *et al*.^19^) shows a good overlap between them.

**Table 2:** Interactions between side-by-side hexamers in the energy minimized cylindrical VP40 ring matrix.

Cylindrical Model	Interaction energy (kcal/mol)^a^	No. of H-bond^b^	Hexamer Separation (nm)^c^
21	−303.56	12.57	5.04
22	−297.18	9.36	5.19
23	−249.89	9.30	5.17
52	−233.05	8.56	4.80
53	−227.34	7.66	4.79
54(a)	−236.75	8.94	4.80
54(b)	−301.05	10.00	4.71

All columns are given for the interactions for a pair of side-by-side hexamers and is the average over all hexamer pairs in the ring. ^a^Interaction energy calculated using the NAMD Energy protocol of VMD. The interaction energy is a total of van der Waals and electrostatic interactions. ^b^Hydrogen bonds per hexamer pair calculated using the Hydrogen Bonds protocol of VMD. ^c^Distance between center of mass of two hexamers.

**Figure 3:**
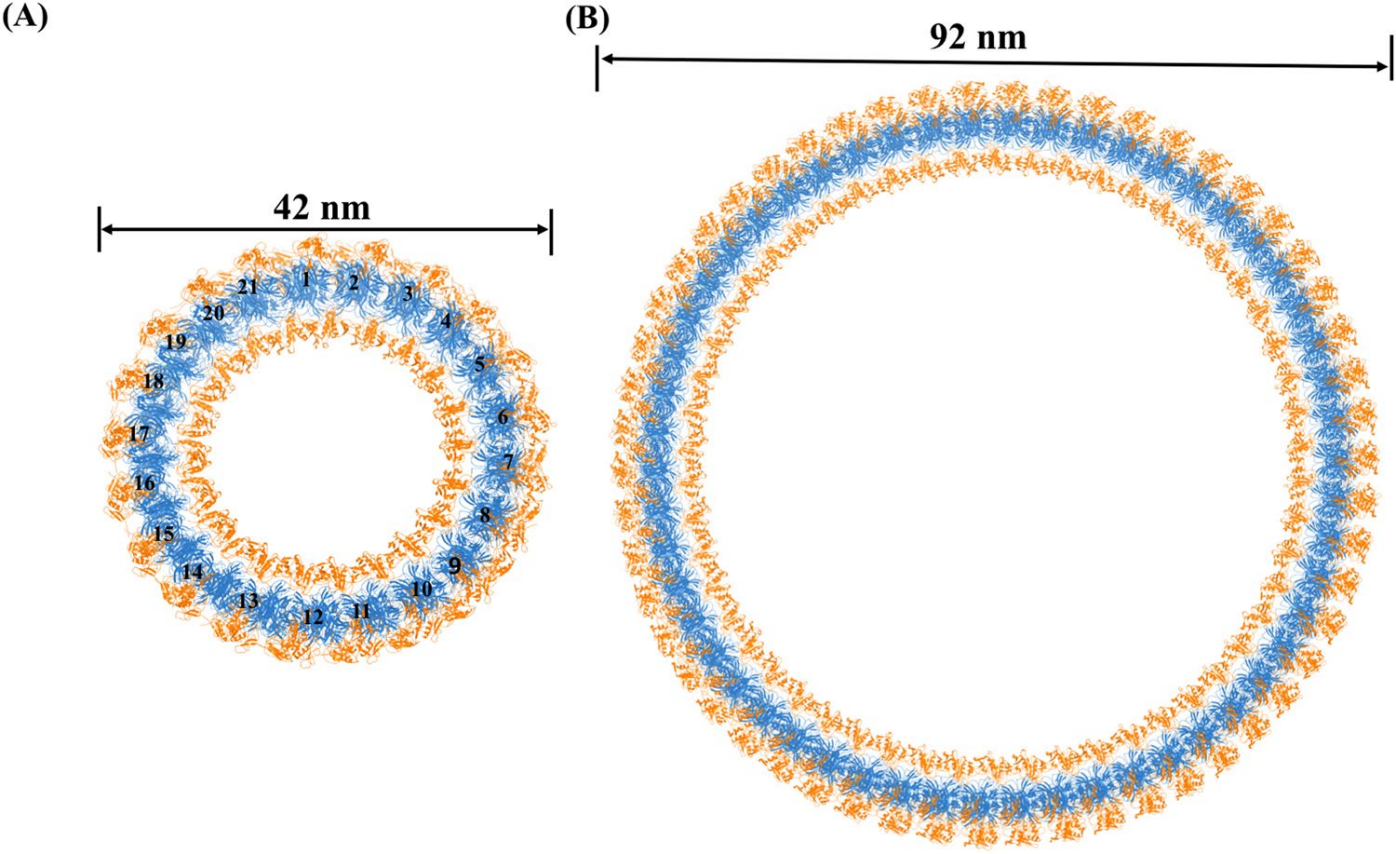
Energy minimized structures of the (**A**) 42 nm diameter VP40 cylindrical matrix consisting of 21 hexamers, (**B**) 92 nm diameter VP40 cylindrical matrix consisting of 54 hexamers [54(b)]. NTD domains are blue and CTD domains are orange. The hexamers are numbered in (**A**).

The energy minimized structures of the 42 nm cylinder containing 21 VP40 hexamers and the 92 nm cylinder containing 54 VP40 hexamers [54(b)] are shown in Fig. 3, and the energy minimized structures of the other 22, 23, 52, 53 and 54(a) cylinders are shown in Figure S1. A pair of hexamers from the three 42 nm diameter rings and the four 92 nm diameter rings were superimposed to understand their structural differences, as shown in Figure S2. The superimposition shows that the hexamer pairs of 21, 22, and 23 (Figure S2A) align well with each other with a low root mean square deviation (RMSD). Similarly, a hexamer pair from each of the cylindrical structures of 52, 53, 54(a) and 54(b) aligned well with each other with a low RMSD [Figure S2B]. This indicates that the VP40 hexamers in the 21, 22, and 23 cylinders arranged in a similar orientation, and the VP40 hexamers in the 52, 53, 54(a) and 54(b) cylinders arranged in a similar orientation to each other. Also, comparison of the hexamer pairs in 21and 54(b) shows that the pairs have very similar side-by-side alignments with minor difference (Figure S3). The difference may be because of the inter-hexamer distance and the difference of the diameters of the rings.

**Figure 4:**
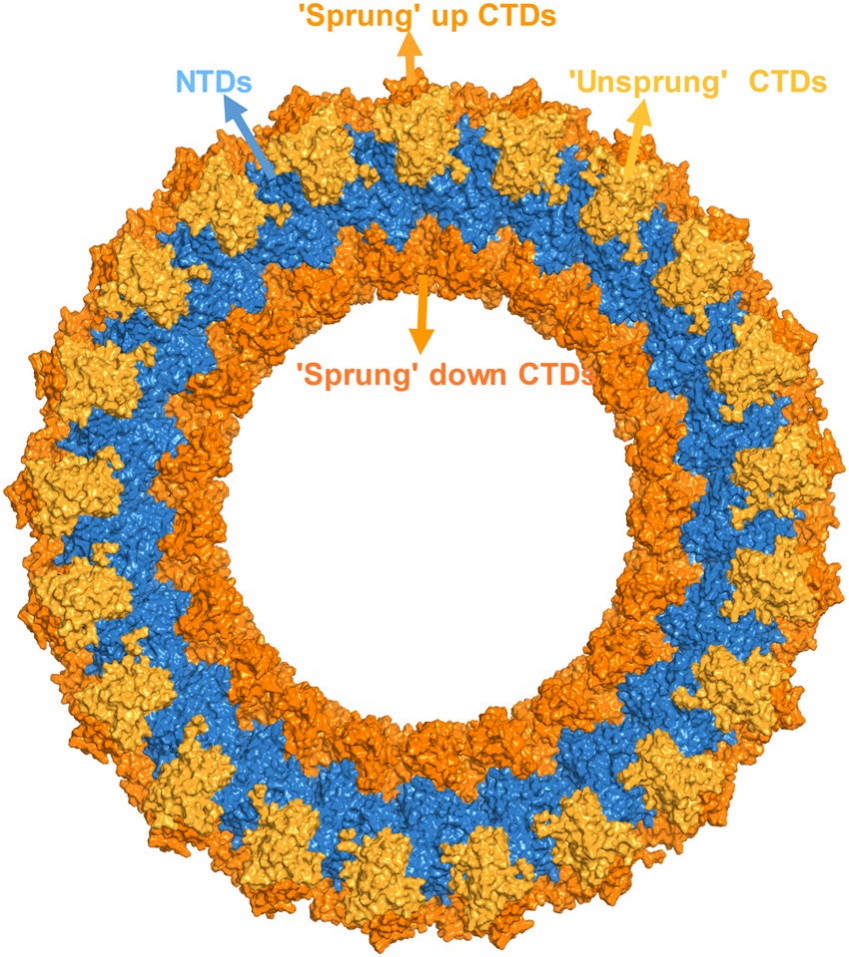
Structure of the cylindrical VP40 matrix The NTDs and “unsprung” CTDs form the central structural core, and sprung up and down CTDs form outer and inner matrix layers. The NTDs are colored in blue, the unsprung central core CTDs are colored in yellow, and the sprung CTDs of the inner and outer matrices are colored in orange.

Our cylindrical models shed light on how the VP40 hexamers can assemble into the cylindrical matrix in the empty (no nucleocapsid) and the native (with nucleocapsid) VLPs. As shown in Fig. 4, there are three structural matrix layers in the cylindrical models: outer, central and inner. As indicated above, the sprung up CTDs form the outer matrix that could facilitate the interaction with the membrane and cytoplasmic tails of GPs. This is consistent with the cryo-EM observations that the outer matrix could make contacts with GPs and this could stabilize the tubular membrane structure^19^. The positioning of the outer CTD domains in our models allows interaction between VP40 and GPs which has also been observed to enhance VLP budding^23,24^. The sprung down CTDs form an inner matrix that could interact with nucleocapsid proteins, which is also consistent with the cryo-EM observations that VP40 can interact with NPs^19,20,25^. The NTDs and the unsprung CTDs create the central core of the matrix and are the main structural scaffold for end-to-end extension into a long filament.

**Figure 5:**
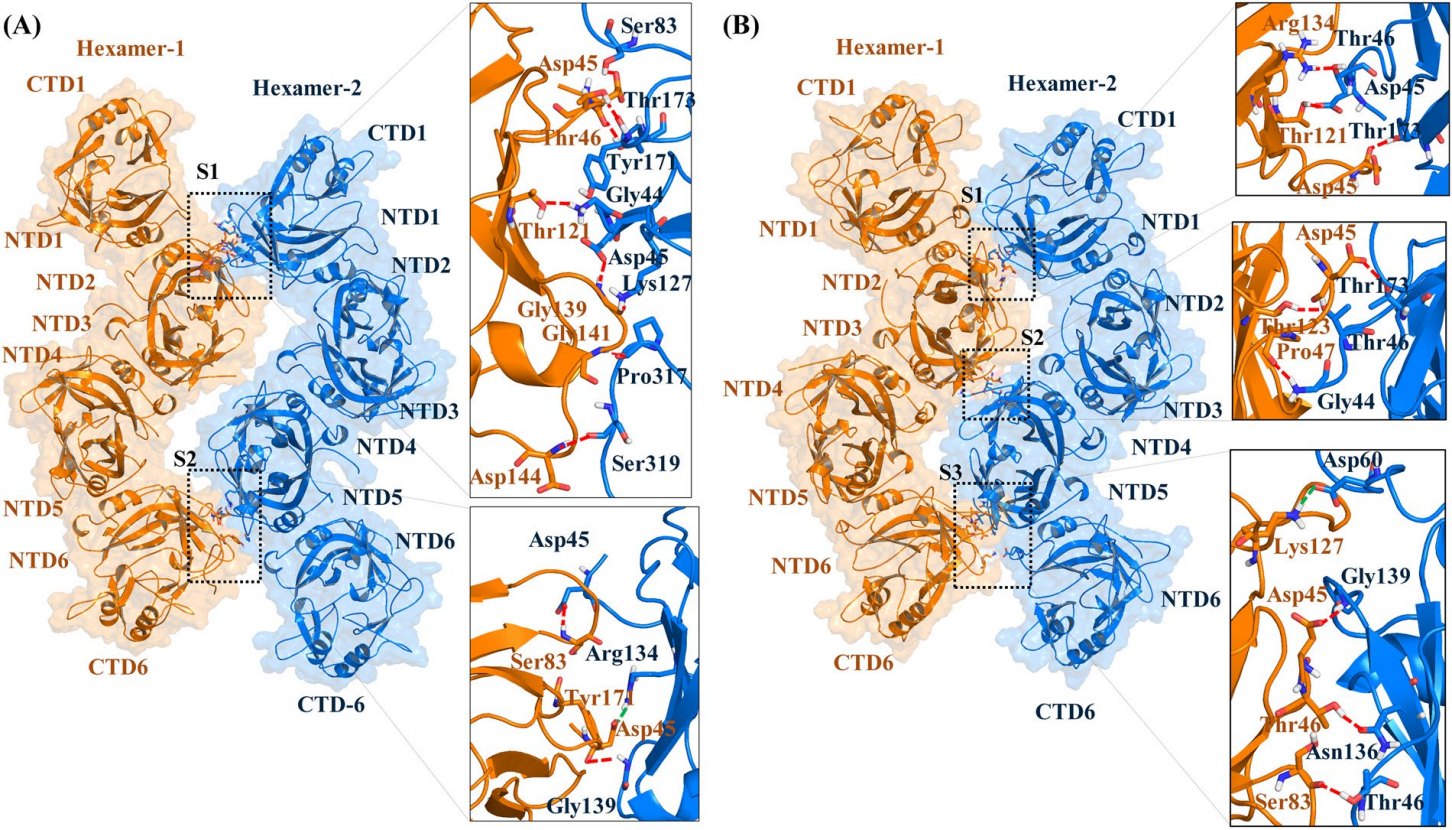
Side-by-side interactions between two hexamers from the energy-minimized VP40 cylindrical matrix of (**A**) Model 21 hexamers and (**B**) Model 54(b). For both (**A,B**), hexamer-1 is colored orange and hexamer-2 is colored in blue. The residues that form hydrogen bonds and salt-bridges at the hexamer-hexamer interfaces are shown in the boxes. Red and green dashed lines respectively show the hydrogen bonds and salt-bridges.

In order to gain insight into the protein-protein interactions at the atomic level, the side-by-side interactions within a pair of hexamers in the energetically stable 21 and 54(b) VP40 cylindrical matrices were investigated. The protein-protein interactions between a pair of hexamers in the 21 and the 54(b) cylinders are shown in Fig. 5A and B, respectively. The analysis of the protein-protein interface shows differences between the 21cylinder compared to the 54(b) cylinder. As computed by the protein interfaces, surfaces, and assemblies (PISA) on-line server^26^, the hexamer-hexamer complex in the 21 cylinder has 1631 and 1594 Å^2^ of solvent-accessible area on hexamer-1 and hexamer-2, respectively, while the hexamer-hexamer complex in the 54(b) cylinder has 1785 and 1811 Å^2^ of solvent-accessible area on hexamer-1 and hexamer-2, respectively. In addition, the change in solvation energy (ΔG) on binding is predicted to favor complex formation by −8.9 kcal/mol for a hexamer-hexamer pair in the 21 cylinder and by −6.2 kcal/mol for a hexamer-hexamer pair in the 54(b) cylinder. The comparisons of the ΔG indicate that the protein-protein interaction in the 21 cylinder is stronger than the 54(b) cylinder, which is due to the differences in the protein-protein interface orientation.

The amino acid interactions in the hexamer-hexamer interface were further examined for both the 21 and 54(b) rings with the use of VMD. The hexamer-hexamer interface in the 21 cylinder has two especially close regions, S1 and S2, as shown in Fig. 5A, with details given in Table S1. A salt-bridge bridge interaction between Asp-45 and Arg-134 occurs in the S2 site [Fig. 5A and Table S1]. The protein-protein interface in the 54(b) cylinder has three especially close regions, S1, S2 and S3, as shown in Fig. 5B, with details given in Table S2. Interestingly, Asp-60, which Bornholdt^14^ found to be important at the NTD-NTD interface that forms the dimer, is also important for stabilizing the side-by-side hexamer arrangement in the ring by forming a salt-bridge with Lys-127 in the S3 site [Fig. 5B and Table S2]. Also, Hoenen *et al*.^27^ found Arg-134 to be important in VP40 RNA binding, and we found that Arg-134 forms a hydrogen bond with Thr-46 that helps stabilize the ring.

The interfacial hydrogen bonds and salt-bridges in the two complexes are analyzed in Table S1 (21-cylinder) and Table S2 [54(b)-cylinder]. The hexamer-hexamer complex of the 21 cylinder has ten hydrogen bonds and a salt-bridge, whereas the 54(b) cylinder has nine hydrogen bonds and a salt-bridge. In addition, many residues in the hexamer-hexamer interface of both cylinders make hydrophobic contacts (not shown). The presence of hydrogen bonds and salt-bridges provide specificity for the correct orientation of the hexamers when forming complexes^28,29^, and the hydrophobic contacts further stabilize the system^30^. In addition, though the two unsprung CTDs from each hexamer are located in the region of the central core matrix, the analysis in Tables S1 and S2 indicates that they contribute little to stabilizing the core. Most of the interface interactions are formed by the NTD domains, confirming that the NTDs are mainly involved in the formation of the central core of the VP40 cylindrical structural matrix. The unsprung CTDs may be involved in the end-to-end binding of hexamers that leads to the VP40 filaments, while the sprung up and down CTD domains interact with membrane and nucleocapsid proteins, respectively^14^.

### Short-term dynamics of the VP40 matrix ring

To investigate the structural dynamics of the VP40 cylindrical matrix, we performed 10 or 15-ns all-atom, implicit solvent molecular dynamics (MD) simulations for the selected cylindrical structures listed in Table S3. To quantitatively understand the stability of the cylindrical ring structures, we computed the root mean square deviations (RMSD) summed over all of the backbone atoms of the cylindrical models relative to their initial structure for the inner and outer matrices as a function of simulation time as shown in Figure S4. For all cylindrical models, the central NTDs and unsprung CTDs have an RMSD compared to their initial structures of approximately 1.2 nm, whereas the sprung CTDs have an RMSD of approximately 1.7 nm, indicating that the sprung CTDs are significantly more flexible than the NTDs. Our simulation results for the flexibility of CTDs and NTDs agrees with the experimental results that the NTDs are rigid, and the sprung up and down CTDs are flexible^14,19^. The functional roles of the sprung CTDs may require enhanced flexibility. For example, the flexibility of the sprung-down CTDs (inner matrix) would allow them to interact with the C-terminus of the nucleocapsid in the 7-nm gap between the nucleocapsid and the VP40. This flexible contact in the gap would also help in providing the flexibility necessary for bending of the viral filaments^19^. In addition, the flexibility of sprung-up CTDs (outer matrix) would help to interact with the plasma membrane^14,19^ and also provide flexibility for bending of the virion. To further understand the conformational changes of the structures, snapshots at different simulation times are shown in Figure S5 for the 42 nm cylinder models and 92 nm cylinder models. In all cases, the ring structure remains intact but changes in the arrangement of individual hexamers can be seen, especially in the inner and outer matrices.

**Figure 6:**
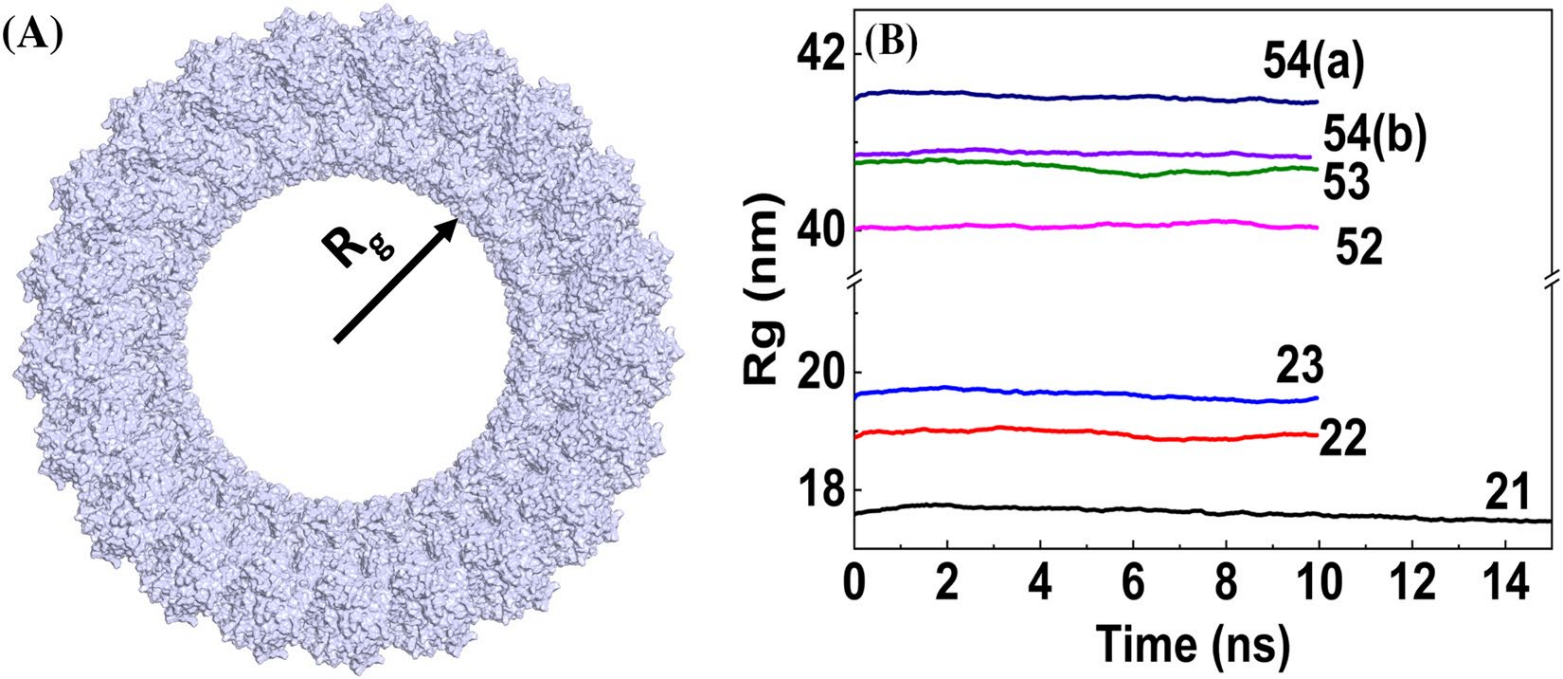
Radius of gyration calculated from the MD simulations for the backbone atoms of the VP40 cylindrical ring matrices as a function of MD simulation time.

To gain additional insight into the structural stability of the VP40 cylindrical matrix, we investigated the radius of gyration (Rg) of the backbone atoms of the cylinders as a function of simulation time as shown in Fig. 6B. The schematic for Rg is shown in Fig. 6A. As can be expected, the Rg is larger for cylinders with more hexamers. In spite of the noticeable RMSD for the cylinders, for all of the 21, 22, 23, 52, 53, 54(a) and 54(b) rings, the Rg remain relatively constant during the MD simulations. This indicates that the cylindrical matrices are structurally stable even though only VP40 is included in our model, and no other viral components.

**Figure 7:**
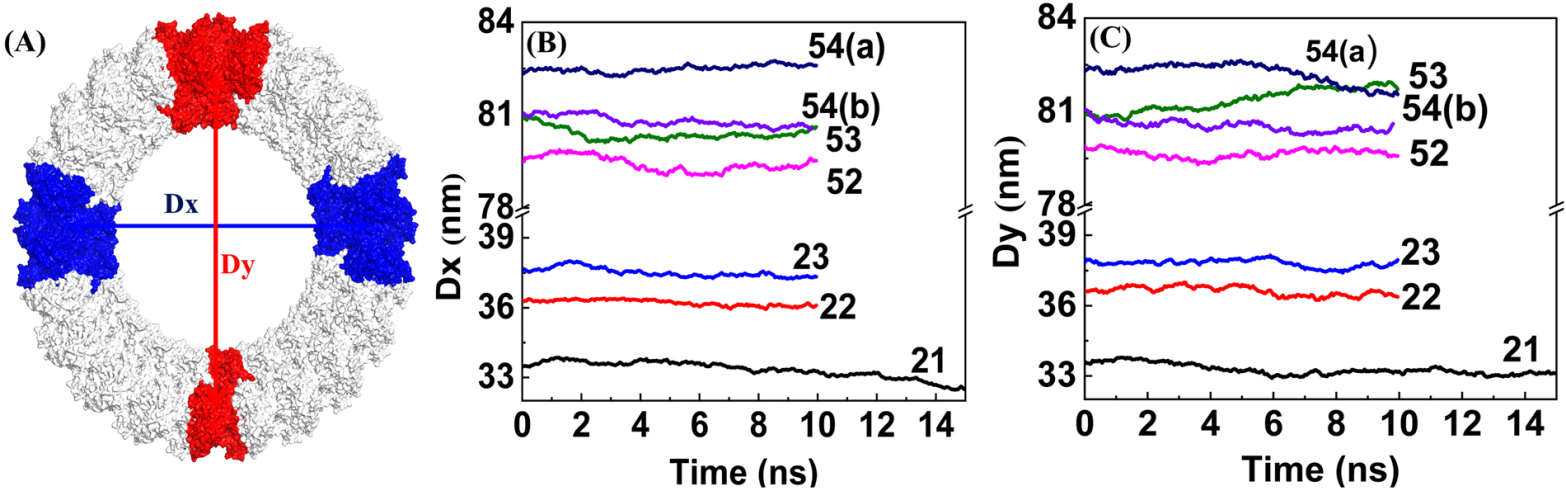
Robustness of circular shape of different size VP40 rings: length of perpendicular axes of rings as a function of MD simulation time. (**A**) Schematic of perpendicular Dx and Dy. (**B**) Dx and (**C**) Dy.

The robustness of the Rg shows that the cylinders do not collapse. We also investigated the stability of the circular shape of the ring with respect to deformation to an elliptical shape. We computed the length of two perpendicular ring axes, Dx (x-axis) and Dy (y-axis), during the MD trajectory. The schematic for the calculation of Dx and Dy is shown in Fig. 7A. The axes Dx was defined for the 21-cylinder, using the hexamer numbering in Fig. 3A, as the distance between the center-of-mass of hexamers 5 and 6 and the center-of-mass of hexamers 15 and 16, while Dy was defined as the distance between the center-of-mass of hexamers 10 and 11 and the center-of-mass of hexamer 21, and the Dx and Dy were calculated similarly for the other ring models. The Dx and Dy as a function of the MD simulation time for all of the cylindrical models are shown in Fig. 7B,C, respectively. As expected, rings with more hexamers have larger Dx and Dy. Figure 7B,C show that for all ring models, Dx and Dy manifest very small fluctuations and remain approximately equal. Along with the stability exhibited by Rg, the relatively constant values of Dx and Dy as a function of time further demonstrate the robustness against deformation by the VP40 ring structures.

**Figure 8:**
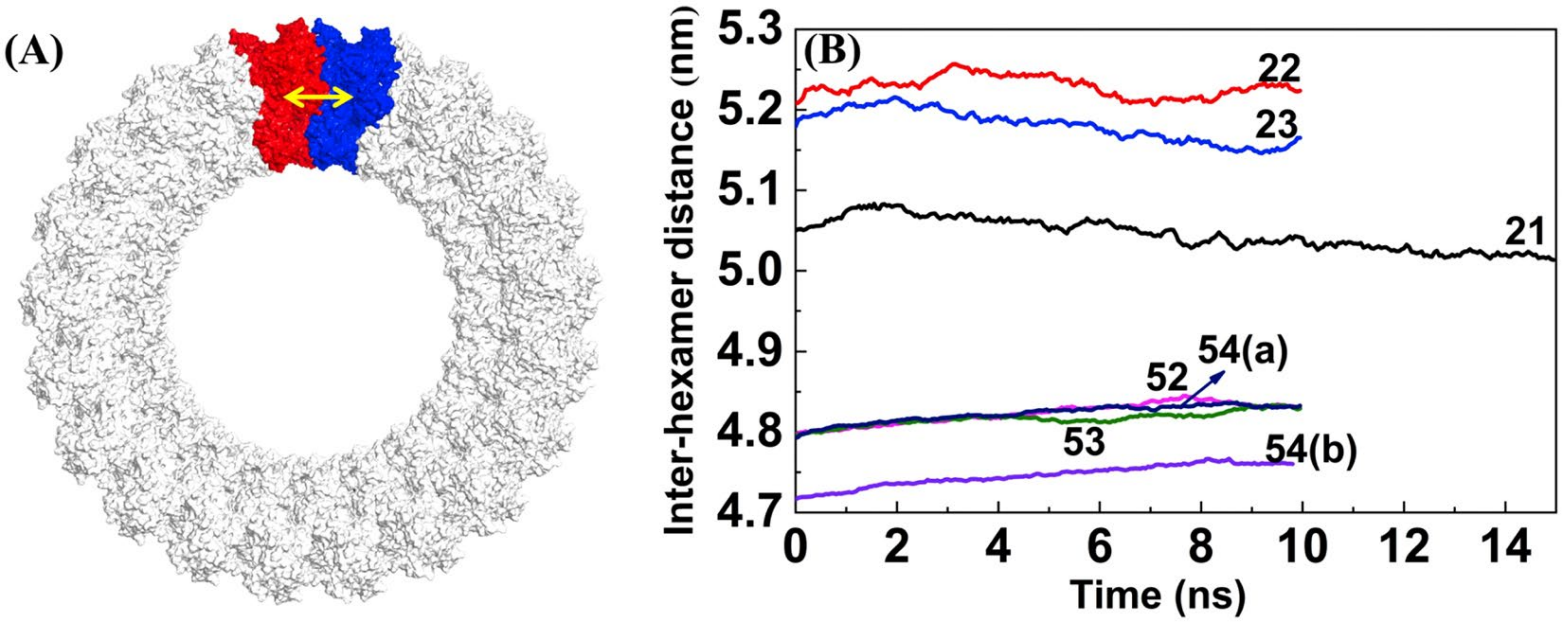
Average side-by-side hexamer-hexamer distance as a function of MD simulation time. (**A**) The yellow arrow highlights the distance between side-by-side hexamers colored in red and blue. (**B**) The average hexamer-hexamer distance as a function of the MD simulation time.

Finally, to investigate the structural flexibility of individual hexamers within the different size rings, we calculated the distance between the center-of-mass of side-by-side hexamers and averaged over all pairs. In Fig. 8, we plot the average hexamer-hexamer distance as a function of the simulation time. The average hexamer-hexamer distance for the 42 nm diameter cylinders fluctuates noticeably, suggesting that there is flexibility of hexamers in the cylindrical ring. On the other hand, the average hexamer-hexamer distance for the 92 nm diameter cylinders displays much smaller fluctuations, indicating a more rigid hexamer-hexamer arrangement within the cylindrical structures. The fluctuations in the 42 nm cylinder may be due to a slightly larger distance between hexamers compared to the 92 nm cylindrical structures.

## Discussion

The Ebola virus matrix protein VP40 is the primary structural protein of the virion and plays a crucial role in viral assembly and the budding of new virus particles from the host cell. VP40 hexamers create a multilayered cylindrical, long filamentous viral matrix. Though the Ebola virus and its nucleocapsid may require other viral proteins (NP, VP24, VP35, and VP40), it has been shown^16–19^ that VP40 alone is capable of forming authentic-looking VLPs without the need of other proteins. Therefore, we investigated the molecular interactions between VP40 hexamers in the cylindrical structural matrix. We have developed 3D computational models for cylindrical VP40 ring structural matrices of both 42 nm (no nucleocapsid) or 92 nm (capable of containing a nucelocapsid) diameter using a protein-protein docking technique. The Ebola virions lacking a nucleocapsid are not infectious, but they provide strong evidence that VP40 alone is capable of self-assembling into a viral structural matrix. The lack of nucleocapsid in the virions formed only with VP40 results in VLPs with much smaller diameter. The inclusion of genetic material, nucleocapsid, and other viral proteins increases the diameter of the VLPs. Sprung VP40 CTDs between the central core of the VP40 matrix and the nucleocapsid provide additional thickness to the infectious VLPs.

Our cylindrical ring models are developed based upon several experimental findings of the cylindrical assembly of VP40, such as the cylindrical diameter, the formation of multilayered structural matrices, and side-by-side hexamer separation. We then performed molecular dynamics computational simulations to investigate the molecular interactions between side-by-side VP40 hexamers in the rings. The relatively short MD simulations were performed to settle the system into a local minimum to optimize the hexamer interactions to explore how the VP40 hexamers are arranged in the viral matrix. The analysis of the simulation results revealed that after some initial changes from the starting structures, the cylindrical structures reached equilibrium configurations that were stable against deformation or collapse. The RMSD and Rg analyses of simulation trajectories indicated that the 42 nm diameter (structural model 21) cylinder is relatively more stable than 92 nm (structural model 54) diameter cylinders. The relatively favorable structural interaction energy of the 21 structural model indicates that the empty (no nucleocapsid) matrix structure can be stable on its own. Though the larger 54(b) structural matrix model without nucleocapsid that we investigated has a higher energy, it can be further stabilized by interactions with the nucleocapsid and other viral proteins in the infectious virus.

We analyzed energetically favorable cylindrical structures and determined the key amino acid residues responsible for the interactions between VP40 hexamers that stabilize the ring structures. Importantly, our simulation studies have demonstrated that the outer and inner structural matrices formed by VP40 sprung CTDs have more structural and dynamical flexibility than the central core matrix, which is consistent with experimental observations. This flexibility of the inner and outer core may be functionally important in facilitating interactions of the VP40 structural matrix with both the virus’ internal nucleocapsid as well as the host cell’s membrane during VLP formation. Such flexibility also allows the nucleocapsid to bend within the envelope and provide virion flexibility. High structural flexibility of virions may be important for viral operation because even a single break would damage the virion and impede its infectiousness^31^.

Overall, the atomistic 3D model of the Ebola cylindrical VP40 matrix presented here can be helpful in understanding how the VP40 hexamer assembles into a cylindrical structure and maintains the structural integrity and flexibility displayed by the virion. We present new details of the side-by-side NTD-NTD interface by which the hexamers stabilize the cylindrical assembly. The importance of the specific hydrophobic, hydrogen bonds, and salt-brides interactions (Fig. 5) in the side-by-side NTD-NTD interfaces between hexamers can be investigated experimentally through mutagenesis studies. The protein-protein interaction sites investigated in this work present potential therapeutic targets for the development of antiviral drugs. For example, small molecule inhibitors targeting the side-by-side protein-protein interactions can interfere with the assembly and stability of the VP40 structural matrix and thus could impede the life cycle of the Ebola virus.

## Methods

### Construction of the cylindrical VP40 matrix

The X-ray crystal structure of the VP40 monomer^10^, dimer, and hexamer^14^ shown in Fig. 1, and cryo-EM and cryo-TM of the VP40 cylindrical matrix of the Ebola virion^19,20^ are reported. The structure of the VP40 hexamer that we used in our cylindrical models was obtained from the X-ray crystal structure (PDB: 4LDD)^14^. Four CTDs^15^ in the hexamer are missing in the X-ray crystal structure, which we added with the Modeller 9.17^32^ software package. We modelled the four CTDs (2, 3, 4, 5; displayed in orange in Fig. 1C) to be consistent with the observations of Bornholdt *et al*.^14^. These four CTDs may be dissociated or become ‘sprung’ away from their respective NTDs to interact with other layers of the filamentous virion. We oriented the middle two CTDs (3, 4) in the hexamer inwards so that they could interact with the virus nucleocapsid, whereas CTDs (2, 5) were oriented on the outward side of the hexamer so that they could interact with the lipid membrane. We used a symmetric side-by-side protein-protein docking approach to construct the model of the cylindrical VP40 ring matrix.

The SymmDock^28^ software was used for the protein-protein docking computational experiment. This docking algorithm creates protein-protein ring complexes with cyclic symmetry by geometry-based docking^28^. Using the information from cryo-EM observations in refs^19,20^, we set the center-of-mass distance between side-by-side hexamers (hexamer-separation) in both the large and small cylindrical complexes to be 5 nm. The experimentally measured diameter of each ring and the hexamer separation distance allows us to calculate an approximate number of hexamers for each ring. With the input of the VP40 hexamer structure and the number of hexamers in the ring (cyclic symmetry order, *C*_*n*_), SymmDock creates a list of complexes with rotational symmetry of order *n* about a symmetry axis. For example, the cylindrical model of symmetric order 21 has 21 VP40 hexamers arranged in cylindrical symmetry. The best cylindrical complexes based on the SymmDock criteria were retained for further investigation. As described more fully in the Results section, the best VP40 cylindrical models were selected based upon visual inspection of protein-protein interactions and the superimposition of the computationally created docking complexes onto the 2D averages of experimentally determined virion tomograms^19^. The stability and dynamics of the selected cylindrical docking complexes were further investigated using molecular dynamics computational simulations.

### Molecular dynamics simulations

All-atom, implicit-solvent molecular dynamics (MD) simulations were performed to minimize the energy and investigate the short-term dynamics of the VP40 rings using the CHARMM22-CMAP force field with torsional cross-terms^33^ with the NAMD 2.12 simulation package^34^. Parameters for the system are given in Table S3. Depending on the number of hexamers in the ring, the number of atoms for the 42 nm ring ranged from 550,431–602,853 and the number of atoms for the 92 nm ring ranged from 1,362,972–1,415,394. The solvent was represented by the generalized Born implicit solvent (GBIS) model with default parameters. For each system, a 50,000-step minimization process was performed using a conjugate gradient^35^. Then the system was heated with a linear gradient of 20 K/ps from 20 to 300 K. At 300 K, the system was equilibrated for 100 ps, followed by a 10 or 15-ns production run with a 2-fs integration time step in the NVT (constant number, volume, and temperature) ensemble. Langevin dynamics with a damping constant of 1/ps was used to maintain the temperature at 300 K. Analysis and visualization of the trajectories were performed with Visual Molecular Dynamics 1.9.4 (VMD)^36^. Figures were prepared using PyMOL-1.8 (http://www.pymol.org/).

### Data availability

All the relevant data are available upon request.

## Electronic supplementary material

Supplementary Information
