# Supplementary material for: A cylindrical assembly model and dynamics of the Ebola virus VP40 structural matrix
Source: Sci Rep. 2018 Jun 27;8:9776. doi: 10.1038/s41598-018-28077-7 (PMC6021417; doi:10.1038/s41598-018-28077-7)
Supplement: Supplementary file 1 — Supplementary Information [file 41598_2018_28077_MOESM1_ESM.pdf]

Supplementary information

A cylindrical assembly model and dynamics of the Ebola virus VP40 structural matrix

Elumalai Pavadai[a*], Bernard S. Gerstman[a,b] and Prem P. Chapagain[a,b]

[a]Department of Physics, Florida International University, Miami, Florida 33199, USA

[b]Biomolecular Science Institute, Florida International University, Miami, Florida 33199, USA

*Corresponding author

E-mail: elumalai.pavadai.a@gmail.com

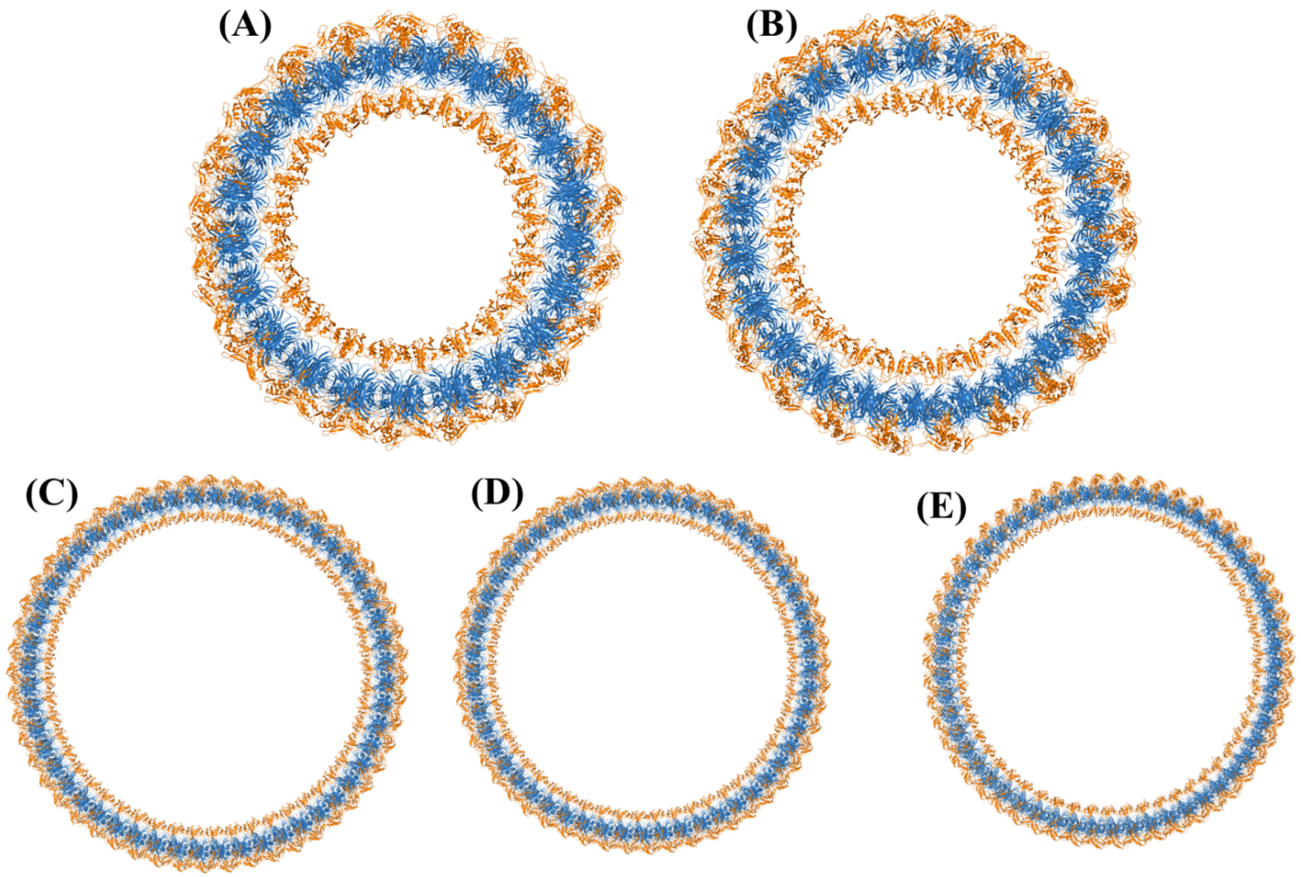

Figure S1. Minimized structures of the VP40 cylindrical ring matrix. Model: (A) 22, (B) 23, (C) 52, (D) 53, (E) 54(a). The proteins are displayed as a ribbon diagram and colored by their domains. NTD domains are sky-blue and CTD domains are orange.

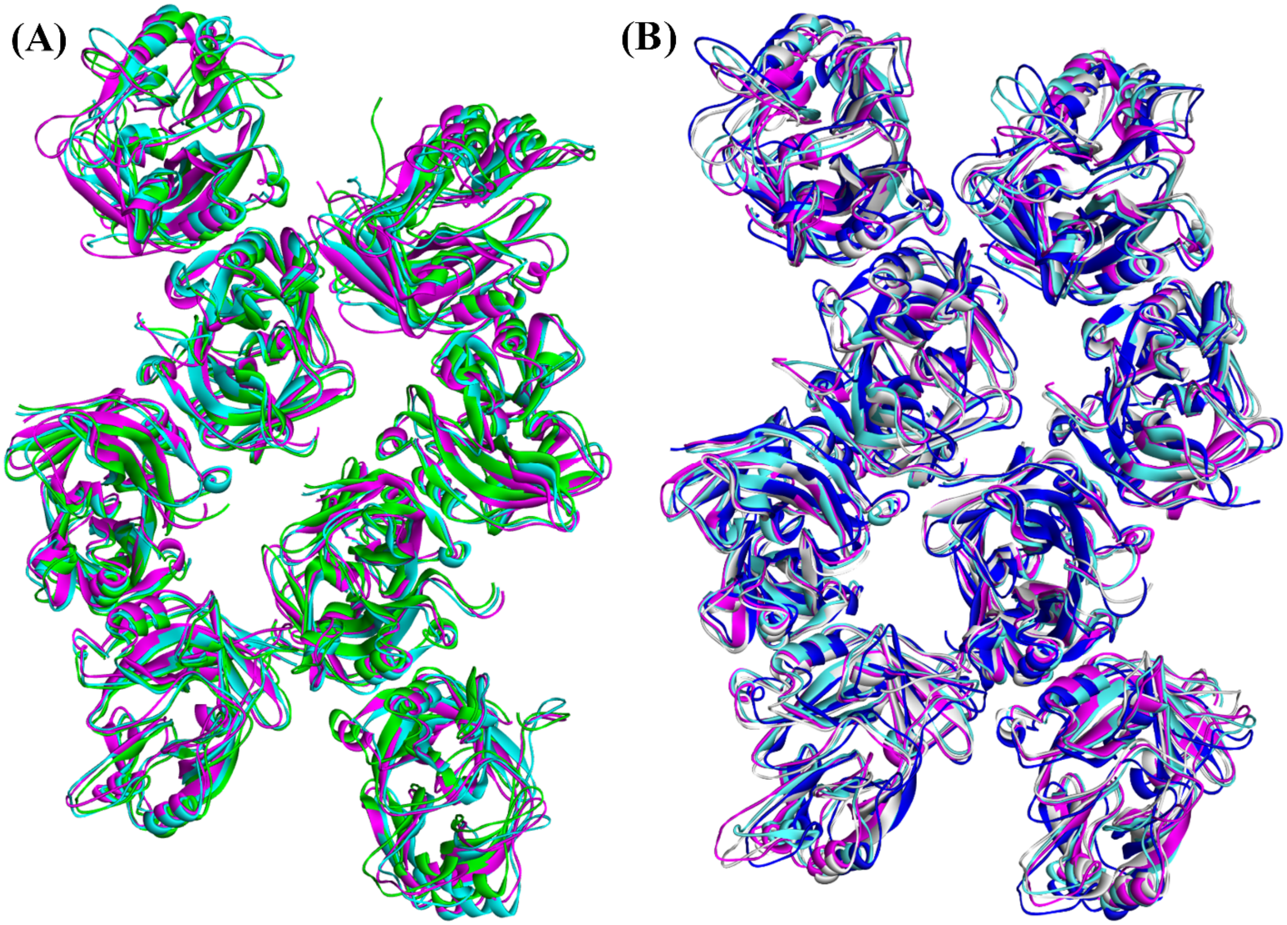

Figure S2. (A) Superimposition of a pair of hexamers from different VP40 cylindrical ring matrix models: (A) 21, 22, 23; (B) 52, 53, 54(a), 54(b). In (A), the pair from 21, 22 and 23 are colored in green, magenta, and cyan, respectively. In (B), the pair from 52, 53, 54(a) and 54(b) are colored in gray, cyan, magenta, and blue, respectively. For clarity, the four "sprung" CTDs on all hexamers are not shown.

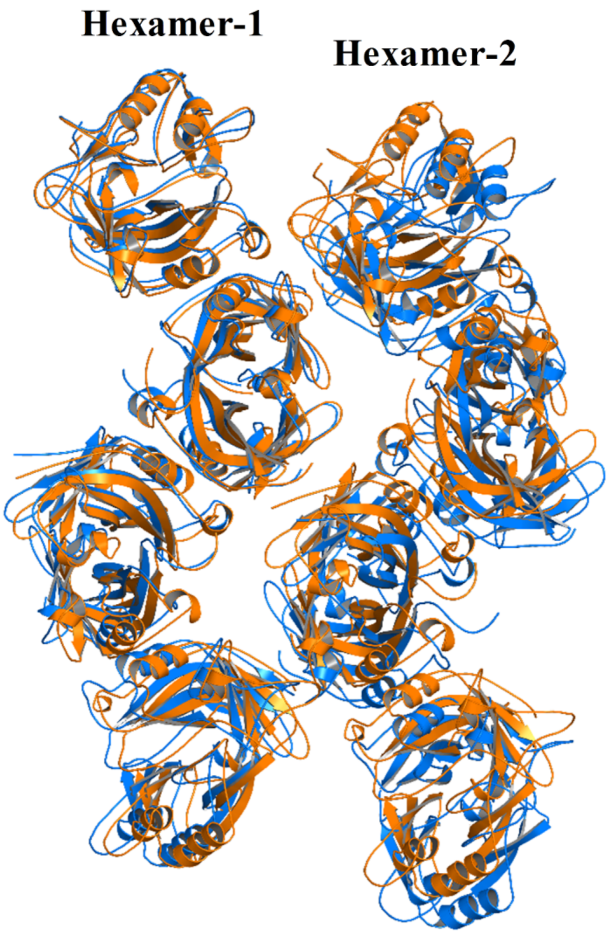

Figure S3. Comparison of a pair of side-by-side hexamers from the 21 and 54(b) model rings. The hexamer pair from the 21 cylinder is colored in blue and the hexamer pair from the 54(b) cylinder is colored in orange. For clarity, the four sprung CTDs (2, 3, 4, 5) of each hexamer that are above and below the main ring are not shown.

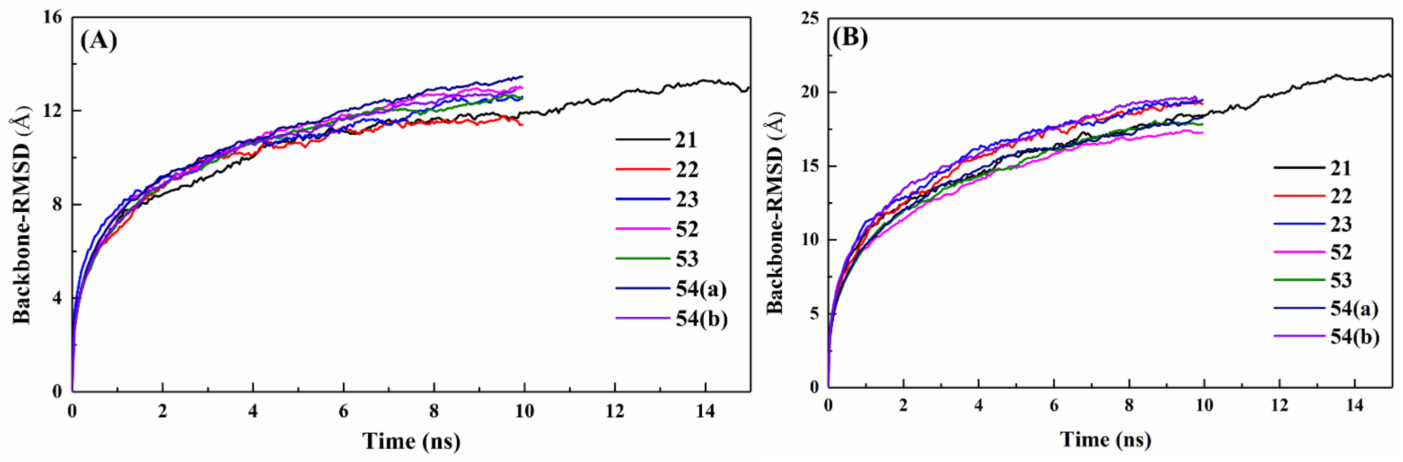

Figure S4. From the MD simulations for the various VP40 cylindrical ring models, root-mean-square deviations (RMSD) calculated for the backbone atoms of (A) NTDs and “unsprung” CTDs, and (B) four “sprung” CTDs. The sprung CTDs have significantly greater RMSD.

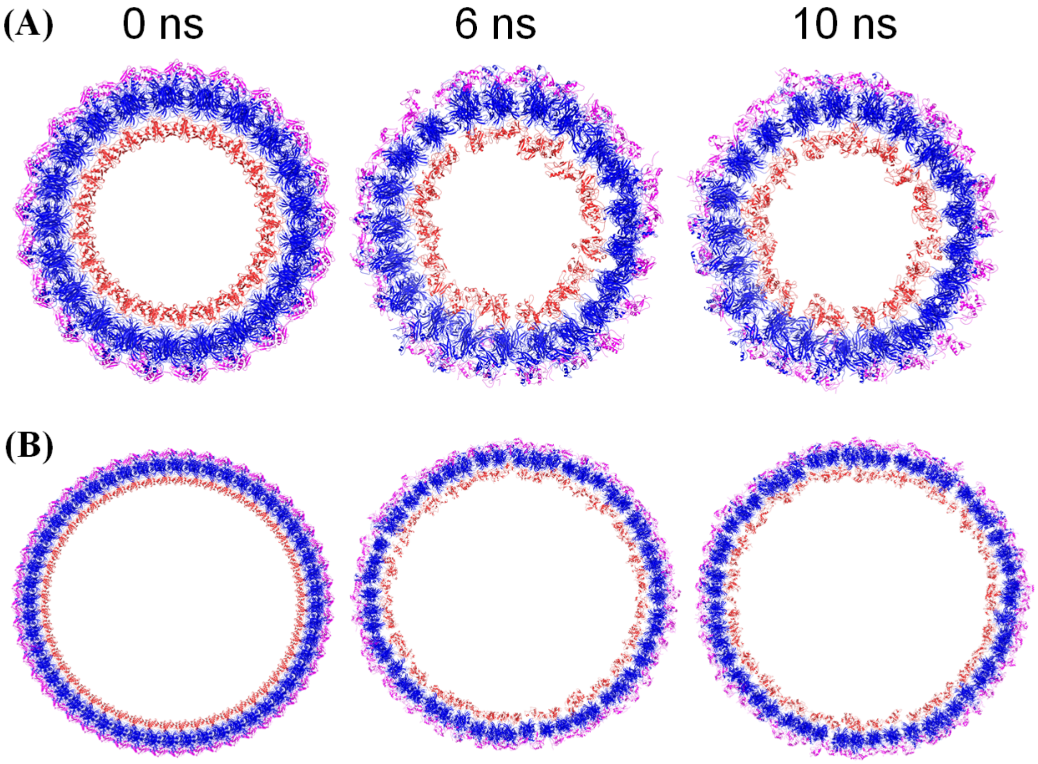

Figure S5. Snapshots of cylindrical structures of (A) 42-nm model with 21 hexamers and (B) 92-nm model with 52 hexamers, at different times during the MD simulation: t=0 (minimized structure), t=6ns, and t=10ns. Central core matrix is blue, outer matrix is magenta and inner matrix is orange.

Table S1. Hydrogen bond and salt-bridge analysis between a pair of side-by-side hexamers in the 42 nm diameter VP40 cylindrical ring matrix containing 21 hexamers. NTD2 refers to the NTD of the second monomer in the hexamer.

Bond[a]	Hexamer-1		Hexamer-2		Distance (Å)	Type[b]
	Residue_Location	Atom	Residue_Location	Atom		
1	Asp-45_NTD2	O	Thr-173_NTD1	OG	2.75	H
2	Thr-46_NTD2	OG1	Ser-83_NTD1	OG	2.77	H
3	Thr-46_NTD2	OG1	Tyr-171_NTD1	O	2.82	H
4	Thr-121_NTD2	OG1	Gly-44_NTD1	N	3.05	H
5	Gly-139_NTD2	N	Asp-45_NTD1	OD1	2.82	H
6	Gly-139_NTD2	O	Lys-127_NTD1	NZ	3.01	H
7	Gly-141_NTD2	N	Pro-317_CTD1	O	2.86	H
8	Asp-144_NTD2	N	Ser-319_CTD1	O	2.88	H
9	Asp-45_NTD6	OD2	Arg-134_NTD5	NH2	2.69	S
10	Ser-83_NTD6	N	Asp-45_NTD5	OD1	2.77	H
11	Tyr-171_NTD6	OH	Gly-139_NTD5	N	3.00	H

[a]1-8 and 9-11 are for S1 and S2, respectively; [b]H and S indicate hydrogen bond and salt-bridge, respectively.

Table S2. Hydrogen bond and salt-bridge analysis between a pair of side-by-side hexamers in the 92-nm diameter VP40 cylindrical matrix containing 54 hexamers [54 (b)]. NTD2 refers to the NTD of the second monomer in the hexamer.

Bond[a]	Hexamer-1		Hexamer-2		Distance (Å)	Type[b]
	Residue_Location	Atom	Residue_Location	Atom		
1	Asp-45_NTD2	O	Thr-173_NTD1	OG1	2.81	H
2	Thr-121_NTD2	OG1	Asp-45_NTD1	OD2	3.00	H
3	Arg-134_NTD2	NH1	Thr-46_NTD1	OG1	2.82	H
4	Asp-45_NTD3	O	Thr-173_NTD4	OG1	2.71	H
5	Pro-47_NTD3	O	Gly-44_NTD4	N	2.82	H
6	Thr-123_NTD3	OG1	Thr-46_NTD4	OG1	3.13	H
7	Asp-45_NTD6	OD2	Gly-139_NTD5	N	1.81	H
8	Thr-46_NTD6	OG1	Asn-136_NTD5	OD1	1.82	H
9	Ser-83_NTD6	O	Thr-46_NTD5	OG1	2.74	H
10	Lys-127_NTD6	NZ	Asp-60_NTD6	OD2	1.67	S

[a]1-3, 4-6 and 7-10 are for S1, S2 and S3, respectively; [b]H and S indicate hydrogen bond and salt-bridge, respectively.

Table S3. Simulated VP40 cylindrical ring systems.

Model system	No. of VP40 Hexamers	Diameter (nm)	Total No. of atoms	Total simulation time (ns)	Temperature (K)
Cylinder-21	21	42	550431	15	300
Cylinder-22	22	42	576642	10	300
Cylinder-23	23	42	602853	10	300
Cylinder-52	52	92	1362972	10	300
Cylinder-53	53	92	1389183	10	300
Cylinder-54(a)	54	92	1415394	10	300
Cylinder-54(b)	54	92	1415394	10	300
